# Identification of Novel Genetic Risk Factors for Cerebral Amyloid Angiopathy

**DOI:** 10.1002/alz70862_110058

**Published:** 2025-12-23

**Authors:** Merve Atik, Joseph S. Reddy, Thuy T. Nguyen, Katie D. Sotelo, Frederick Q Tutor‐New, Minerva M. Carrasquillo, Jonathan Graff‐Radford, Neill R. Graff‐Radford, Kejal Kantarci, Michael A. DeTure, Dennis W. Dickson, Mariet Allen, Nilüfer Ertekin‐Taner

**Affiliations:** ^1^ Mayo Clinic Graduate School of Biomedical Sciences, Florida, FL USA; ^2^ Mayo Clinic, Jacksonville, FL USA; ^3^ Mayo Clinic in Florida, Jacksonville, FL USA; ^4^ Department of Neurology, Mayo Clinic, Rochester, MN USA; ^5^ Department of Neurology, Mayo Clinic, Jacksonville, FL USA; ^6^ Department of Radiology, Mayo Clinic, Rochester, MN USA; ^7^ Mayo Clinic Florida, Jacksonville, FL USA

## Abstract

**Background:**

Cerebral amyloid angiopathy (CAA), characterized by the accumulation of amyloid‐beta in the cerebrovasculature, affects blood vessel integrity leading to brain hemorrhages and an accelerated cognitive decline in Alzheimer’s Disease (AD) patients. Our previous genome‐wide association study (GWAS) identified a *LINC‐PINT* splice variant associated with lower CAA levels in individuals without the *APOE*e4 allele and higher levels of *LINC‐PINT* expression in the brain of AD donors. In this study, we expand our GWAS to include additional donors with AD and those lacking significant AD neuropathology (non‐AD), with available CAA scores.

**Method:**

In our prior study we assessed 853 AD donors. We expanded this by addition of genetic data from 550 AD and 502 non‐AD donors from the Mayo Clinic Brain Bank, scored for CAA. We performed QC and imputation (TOPMED) of all datasets. We conducted GWAS in AD only (*N* =  1,363), non‐AD only, and all donors (*N* = 1,865) by testing imputed variant dosages for association with square root transformed CAA using linear regression, adjusting for relevant covariates. To assess associations in the context of major CAA risk factors, we performed interaction analysis with *APOEe4* presence and sex; and pursued stratified analyses.

**Result:**

Variants at the *APOE* locus were identified as the most significant in our study. In addition, several other variants approached genome‐wide significance after adjusting for AD neuropathology (Braak stage and Thal phase). **T**he *LINC‐PINT* splice variant remained associated with lower CAA scores in AD donors without the *APOEe4* risk allele. To enhance the robustness of our findings, we are pursuing further expansion of our study cohort to include other available datasets. To explore putative functional consequences of key variants we are collecting peripheral gene expression measures in participants from Mayo Clinic with neuroimaging measures including microhemorrhages.

**Conclusion:**

We expect this study will provide further insights into the genetic architecture underlying risk for CAA, both in the context of significant AD pathology, and without. Characterization of genetic variants and their functional outcomes may lead to new avenues of research aimed at identifying biomarkers and therapies to treat CAA, a common co‐pathology in those with and without AD.

